# Inspection of chicken wings and legs for animal welfare monitoring using X-ray computed tomography, visual examination, and histopathology

**DOI:** 10.1016/j.psj.2023.103403

**Published:** 2023-12-27

**Authors:** Kacper Libera, Roozbeh Valadian, Patiharn Vararattanavech, Sri Nithya Dasari, Timothy J. Dallman, Erik Weerts, Len Lipman

**Affiliations:** ⁎ Institute for Risk Assessment Sciences (IRAS), Utrecht University, 3584 CM Utrecht, The Netherlands; † Department of Computational Imaging, Centrum Wiskunde & Informatica, 1098 XG Amsterdam, The Netherlands; ‡ Division of Pathology, Department Biomolecular Health Sciences, Faculty of Veterinary Medicine, Utrecht University, 3584 CL Utrecht, The Netherlands

**Keywords:** animal well-being, limb fracture, fracture detection, X-ray computed tomography, CT

## Abstract

In broiler chickens, fractures of wings and legs are recorded at poultry slaughterhouses based on the time of occurrence. Prekilling (**PRE**) fractures occur before the death of animal, so the chicken was still able to experience pain and distress associated with the injury (an animal welfare issue). Postkilling (**POST**) fractures occur when the chickens are deceased and fully bled-out and consequently unable to feel pain (not an animal welfare issue). Current practice dictates that fractures are recognized visually and recorded by the animal welfare officers as mandated by European Union and/or national regulations. However, new potential monitoring solutions are desired since human inspection suffers from some significant limitations including subjectivism and fatigue. One possible solution in detecting injuries is X-ray computed tomography (**CT**) scanning and in this study we aim to evaluate the potential of CT scanning and visual inspection in detecting limb fractures and their causes. Eighty-three chicken wings and 60 chicken legs (*n* = 143) were collected from a single slaughterhouse and classified by an animal welfare officer as PRE, POST or healthy (**HEAL**). Samples were photographed and CT scanned at a veterinary hospital. The interpretation of CT scans along with photographs took place in 3 rounds (1. CT scans only, 2. CT scans + photographs, 3. photographs only) and was performed independently by 3 veterinarians. The consistency of the interpretation in 3 rounds was compared with the animal welfare officer's classification. Furthermore, selected samples were also analyzed by histopathological examination due to questionability of their classification (PRE/POST). In questionable samples, presence of hemorrhages was confirmed, thus they fit better as PRE. The highest consistency between raters was obtained in the 2nd round, indicating that interpretation accuracy was the highest when CT scans were combined with photographs. These results indicate that CT scanning in combination with visual inspection can be used in detecting limbs fracture and potentially applied as a tool to monitor animal welfare in poultry slaughterhouses in the future.

## INTRODUCTION

The modern poultry sector is distinct from other branches of animal production in terms of the rapid, large-scale and high-density production, great intensification and automation at almost every stage of primary production (Gržinić et al., 2023). Chicken flock sizes often exceed thousands of animals; thus the animal welfare status of the single animal is often overlooked. Animal welfare has been previously defined and is often expressed in terms of 5 freedoms of the animals (Mellor, 2016) including freedom from pain, injury, and diseases. In the European Union (**EU**) as a standard operational procedure, animal welfare inspection is obligatory at the slaughterhouse based on the EU regulations (Council Directive, 2007/43/EC). EU law obligates business operators to introduce procedures to monitor different aspects of their performance including animal welfare (EU Regulation 1099, 2009). However, the national authorities specify the details about this inspection (e.g., frequency, sample size, and critical limits). For example, in the Netherlands, the Dutch food safety authorities (**NWVA**—Nederlandse Voedsel-en Warenautoriteit) sets the threshold for prevalence of injuries in poultry slaughterhouse at 2% of the chickens affected within the batch (NVWA Report, 2022). If the batch does not comply with these requirements, then the poultry producer and/or the catching team can receive a fine.

Broiler chickens are particularly susceptible to welfare issues including injuries such as wing fractures. This is due to their relatively small size, fragile skeletal system (as they are still juveniles at the time of slaughter), and the way they are handled before being processed (Kittelsen et al., 2015). There are 2 types of fractures frequently diagnosed in poultry slaughterhouse. The first type of injury, which occurs before killing, called prekilling (**PRE**), can be caused by improper catching, loading, transportation, or unloading/handling in the slaughterhouse (Kittelsen et al., 2015). However, it is important to note that even if the catching teams use catching methods that are more considerate of animal welfare (e.g., catching in an upright position, not catching by legs) or reduce the speed of the loading, still wing and leg fractures can occur (Kittelsen et al., 2018; Cockram et al., 2020). Similarly, in mechanical loading (e.g., a dedicated harvester, with no direct animal-human interaction) wing fractures are still reported (Monch et al., 2020). Therefore, wing or leg injuries will still occur even when the best practices are applied, but at a lower frequency. Nevertheless, PRE fractures cause significant distress and suffering for the animal, as they are still alive and able to experience emotions such as pain and fear. Furthermore, such physical trauma will induce a local inflammatory response, which will lead to microscopically observable and potentially also macroscopically evident morphologic changes of the tissue, such as limb swelling, discolorations, hemorrhages/petechiae, and/or bruises. Conversely, there are fractures that occur after the animals are dead and fully bled-out termed postkilling (**POST**). While these fractures no longer pose an animal welfare concern, they do create an important food quality issue for the plant operator. Carcasses with broken wings are difficult to sell for human consumption. This type of fracture can often be attributed to faulty or suboptimal operation of the slaughterhouse machinery. For instance, rubber fingers can cause mechanical damages to the carcass during defeathering (Shung et al., 2022). Regardless of the fracture type, it is important to detect and remove broken limbs from the production line, since these parts are not suitable for human consumption. According to EU Regulation 627 (2019), the official veterinarian must declare fresh meat unfit for human consumption if it, among others, indicates pathological or organoleptic changes (EU Regulation 627, 2019) and any visible limb damage including fractures can be considered as pathological or organoleptic change, for example, subdermal/intramuscular hematomas causing reddish coloration of the wing/leg skin. Furthermore, consumers show a preference at retail for uniformity of meat color and discoloration in the chicken wing or leg due to the fracture will generally be discouraging for consumers (Adamski et al., 2017).

Automated detection and distinction of PRE and POST fractures would facilitate an increase in the numbers of inspected animals and could reduce the possible inaccuracies of human visual inspection, which can negatively affect the consistency and repeatability of the inspection. Furthermore, the detection of POST fractures indicates to the plant operator that machinery adjustments are required to achieve a lower incidence of POST and a higher number of wings that can be sold. The benefits of such system would enhance animal welfare monitoring, increase time- and cost-efficiency as well as enhancing the accuracy of inspection. The optimal solution should be reliable, objective, and possible to implement in the slaughterhouse environment, safety of use must be also taken under consideration. One possible tool that may meet these requirements is X-ray computed tomography (**CT**). In brief, X-ray CT scanners pass X-ray photons through an object, such as a patient or sample, at multiple angles, typically over 360 degrees. The variations in density across different tissues, such as muscle and bone, alter the intensity of photons that successfully pass through the object and reach the detector. The scanner then generates a series of 2D images, called projections, at each angle for the corresponding object, showing the density information of the object at that specific angle. These projections are then processed to generate a 3D dataset/volume allowing for the creation of sequential images, or slices, of the object's internal structure (Hermena and Young, 2022).

To the best of our knowledge, the use of CT scanning has not been reported before in terms of recognizing limb fractures in broiler chickens. Therefore, the main aim of this study was to evaluate the potential of CT scanning and visual inspection in identifying wings and legs fractures in broiler chickens to distinguish between PRE and POST fractures. This information is essential to compare and potentially raise animal welfare monitoring standards between slaughterhouses and estimate the economic losses associated with each type of limb fracture. We also present an analysis of animal welfare inspection reports from the collaborating slaughterhouse.

## MATERIALS AND METHODS

### Analysis of Animal Welfare Inspection Reports

Data regarding animal welfare-related findings recorded between January and March 2022 from a slaughterhouse located in the central region of the Netherlands were obtained. The incidence of animal welfare issues was recorded independently by 2 animal welfare officers during a 2-min inspection (equal to 360 carcasses inspected) of a flock. The data exploration, analysis of data distribution and normality (Shapiro-Wilk test, statistical significance set at *P* value = 0.05), and calculating descriptive statistics (mean and standard deviation, median) were performed using SPPS IBM statistics version 28.0.1.0 (IBM Software, New York, NY). The spectrum of analysis included country of flock origin, average flock size and the most prevalent animal welfare-related findings.

### Sample Collection

A total of 143 chicken (Ross 308) wings and legs were collected from the slaughterhouse as described above, which uses a CO_2_ chamber for stunning and an automatic system for bleeding. The wing typically consisted of humerus, radius, ulna, carpus, and manus bones with surrounding musculature and skin commercially, referred to as drumette, wingette, and tip. The femur, tibiotarsus, and fibula with surrounding musculature and skin was considered as a leg commercially referred to as a thigh-drumstick. Samples were collected over 3 d, with 38 samples (24 wings and 14 legs) collected on the first day, 39 samples (23 wings and 16 legs) collected on the second day, and 66 samples (36 wings and 30 legs) collected on the third day. The wings and legs were collected by the slaughterhouse staff at the end of the processing line. The samples were divided and put in 3 different containers based on the decision of the animal welfare officer. The decision possibilities of the animal welfare officer were: 1) PRE fracture, 2) POST fracture, 3) healthy (**HEAL**) wing/leg (no fracture/injury). Briefly, the basis for animal welfare decision was: 1. if the sample had an open fracture (the bone protruded through the skin) or a non-natural bone/joint alignment and bruises, it was considered as PRE 2. if the sample had an open fracture or non-natural bone/joint alignment with no bruises, then it was considered as POST and 3. if the sample had no open fracture or non-natural bone/joint alignment nor any bruises, it was considered as HEAL. The samples were transported in a cooling box at +4°C to the lab within 1 h and kept overnight in the refrigerator for further analysis the next day.

### Samples Handling and Identification

Each sample was given a unique label and was stored in separate transparent plastic bag. In addition to the unique sample number, each label also included the category assigned by the animal welfare officer (PRE/POST/HEAL). Photographs from both sides were taken (camera with 12 megapixels, resolution 3,024 × 4,032 pixels) to allow easy identification and documentation.

### Fracture Characterization

Each PRE and POST sample was described in detail regarding the injury location using CT scans and visual examination. These descriptions included the type of limb (wing/leg), affected bones/joints, location (distal, midshaft, proximal), type of injury (comminuted, oblique, transverse fracture, or dislocation).

### CT Scanning

All samples were scanned using a SOMATOM Definition AS CT scanner (Siemens Healthineers, the Hague, the Netherlands) at the veterinary hospital of Utrecht University. The scanner's software presets were utilized to suggest the optimal combination of voltage and current based on sample size, thickness, and density. In most cases, the parameters were set to 120 or 80 kV and 80 to 150 mA, slice thickness = 0.6 mm. Legs and wings were scanned separately. The samples from each day were scanned either on the same day or the following day depending on the availability of CT scanning unit. Approximately 8 to 12 wings or 3 to 5 legs were put together on the (patient) table and scanned at once (batch). Samples from the same day were selected randomly to form a batch, therefore in most cases the batch was combination of POST, PRE and HEAL samples.

### Interpretation of CT Scans

**Figure 1:**
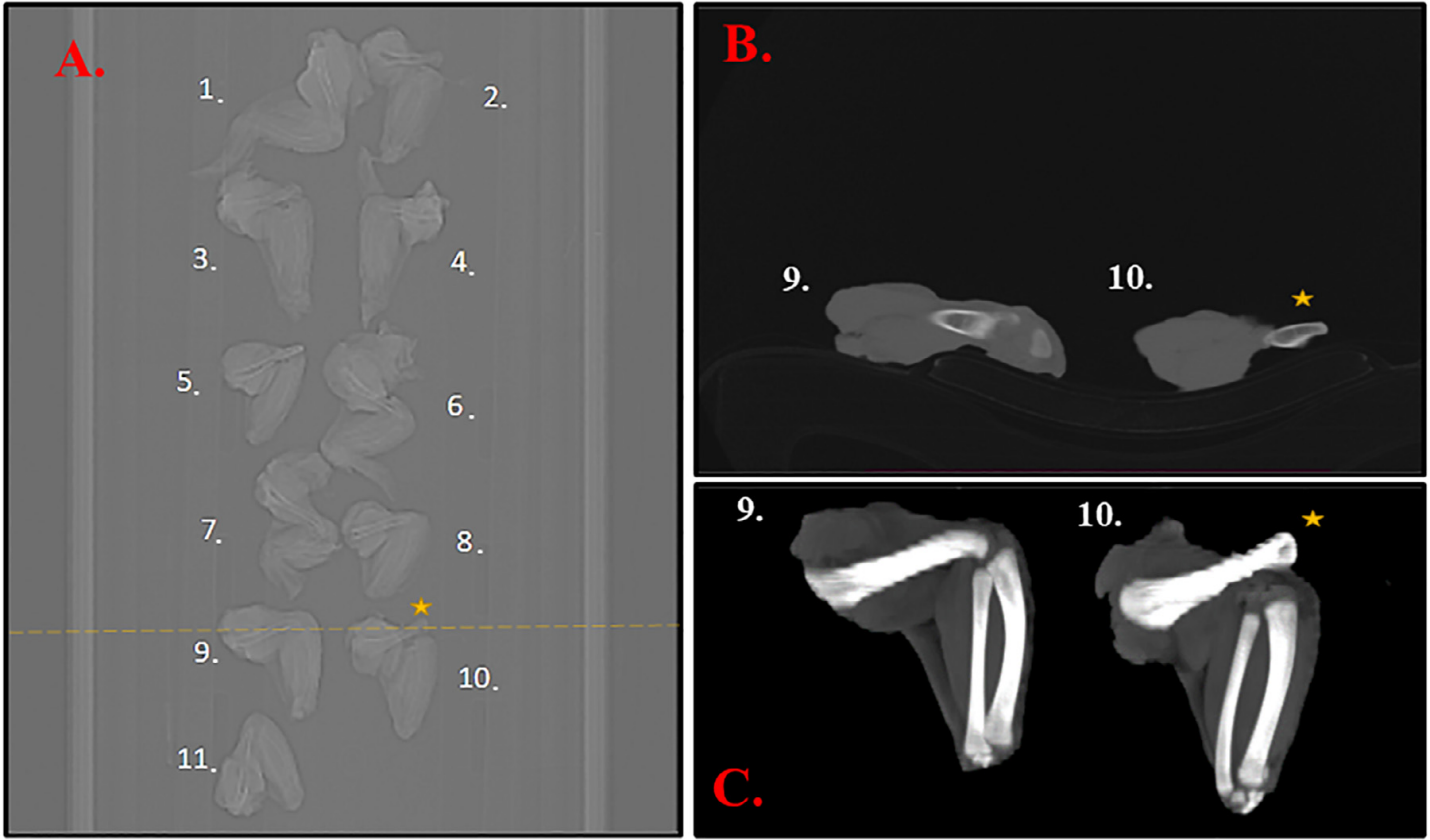
An example of wing CT images. The panel A presents a topogram, the panel B. is the reconstructed volume, while the panel C shows the maximum intensity projection. The yellow dashed line in A indicates the location of the cross-section in B. Sample #9 has no injuries, while sample #10 was recognized with elbow dislocation. The star sign (⭐) indicates the exact location of the injury.

**Figure 2:**
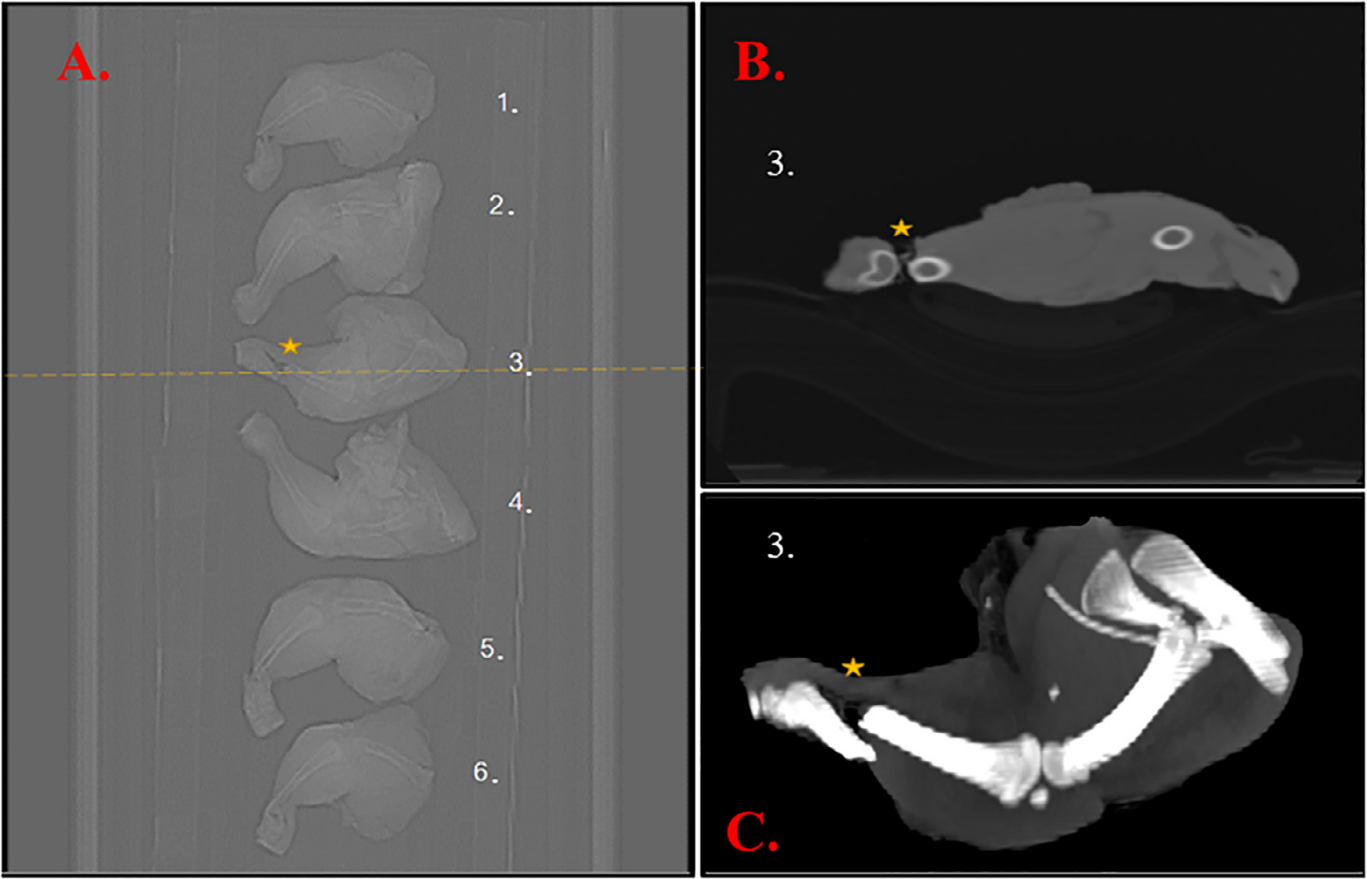
An example of leg CT images. The panel A presents a topogram, the panel B is the reconstructed volume, while the panel C shows the maximum intensity projection. The yellow dashed line in A indicates the location of the cross-section in B. Sample number #3 was recognized with tibiotarsus fracture. The star sign (⭐) indicates the exact location of the injury.

The samples (*n* = 143) were  divided into 21 batches labeled alphabetically from A to U. The scans, saved in DICOM format, of all examined batches can be found in the Supplementary materials (https://surfdrive.surf.nl/files/index.php/s/mLPzwZ7OEQ8r0C4). The scan was defined as either a projection (2D grayscale images at different angles) or a reconstructed slices/volume (2D grayscale images/slices at different Z location) (examples given in Figure 1, Figure 2) saved in DICOM format. The interpretation of the scans was performed by 3 independent general veterinarians. The interpreters used RadiAnt DICOM VIEVER software (Medixant, Poznan, Poland). The interpretation of CT scans was blinded, with the veterinarians not knowing either the label of the sample nor the batch/day of the scanning. The veterinarians worked independently with no possibility of consultation.

The images were examined 3 times (1st, 2nd, and 3rd round) by each of the 3 interpreters.

In the 1st round, 3 veterinarians independently assessed each CT scans of the leg/wing samples and classified each based on the type of sample (wing/leg) and the presence of injury (yes/no) and its type (PRE or POST). In the 2nd round, the same veterinarians assessed the same set of randomized CT scans accompanied by corresponding photographs of both sides of the leg/wing and in the 3rd round only photographs were used during interpretation. Three rounds took place on 3 different days with an approximately 1-mo interval between the 1st and 2nd round and 7-mo interval between the 2nd and 3rd round.

### Level of Agreement Between Raters

The level of agreement between the 3 veterinarians (using either CT scans, CT scans + photographs or photographs only) and the animal welfare officer from the slaughterhouse was calculated and expressed as Fleiss’ kappa. Fleiss' kappa is a measure of inter-rater agreement used to determine the level of agreement between 2 or more raters, when the method of assessment is measured on a categorical scale (Fleiss et al., 2003). This method compares observed accuracy of rating with an expected accuracy (random chance). Reliability using Fleiss` kappa can be interpreted as poor (<0), slight (0.01–0.20), fair (0.21–0.40), moderate (0.41–0.60), substantial (0.61–0.80), or almost perfect (0.81–1.00) (Landis and Koch, 1977). The value of Fleiss’ kappa was calculated separately for the 1st, 2nd, and 3rd round. The statistical significance was set at *P* value = 0.05. In Fleiss` kappa statistical inference the null hypothesis states the agreement between the raters is no better than chance, therefore in case of *P* value <0.05 it can be concluded that the level of agreement between raters was significantly different (e.g., higher) than if the raters would randomly allocate labels to the samples (random chance). The calculations were performed using SPPS IBM statistics version 28.0.1.0 (IBM Software, New York, NY).

### Histopathology

In total 8 tissue samples (4 legs and 4 wings) were examined histopathologically to further characterize tissue morphology and lesions in order to determine whether these matched the conclusions based on observations from visual examination and CT scanning. The samples were stored in the freezer (-18°C) for approximately 1.5 mo before processing. The classification of these samples was based on the visual examination of the slaughterhouse's animal welfare officer, who labeled 1 leg and 1 wing as healthy (**HEAL**), 1 leg and 1 wing as PRE, and 1 leg and 1 wing as POST. There were 2 additional samples, also 1 leg and 1 wing, with unsure (**QUEST**) classification, since these samples did not look healthy, but lesions could not well be characterized based on visual inspection (category QUEST was not further used in the visual and CT scan examinations). The categorization (HEAL, PRE, POST, QUEST) was based on visual examination with the following terminology: no changes (**NCh**); blood-stained (**Bs**); petechiae/bruises/hematoma (**PCh**); non-natural bone/joint alignment (**Na**); open fracture/dislocation (**OFd**); *lesions were reported in a mild form.

For histopathologic evaluation, tissues were fixed in 10% formalin for at least 24 h, dehydrated and embedded in paraffin. Four micrometer tissue slides were cut and stained with hematoxylin and eosin (**HE**) according to standard laboratory procedures. Slides were evaluated and tissue changes were summarized with the following terminology: no changes (NCh); hemorrhage (**H**); necrosis (**N**); granulocytic infiltration (**GrI**); mononuclear infiltration (**MoI**); fibrosis (**F**); Increased Intracellular space (**Is**).

## RESULTS

### Analysis of Animal Welfare Inspection Reports

**Figure 3:**
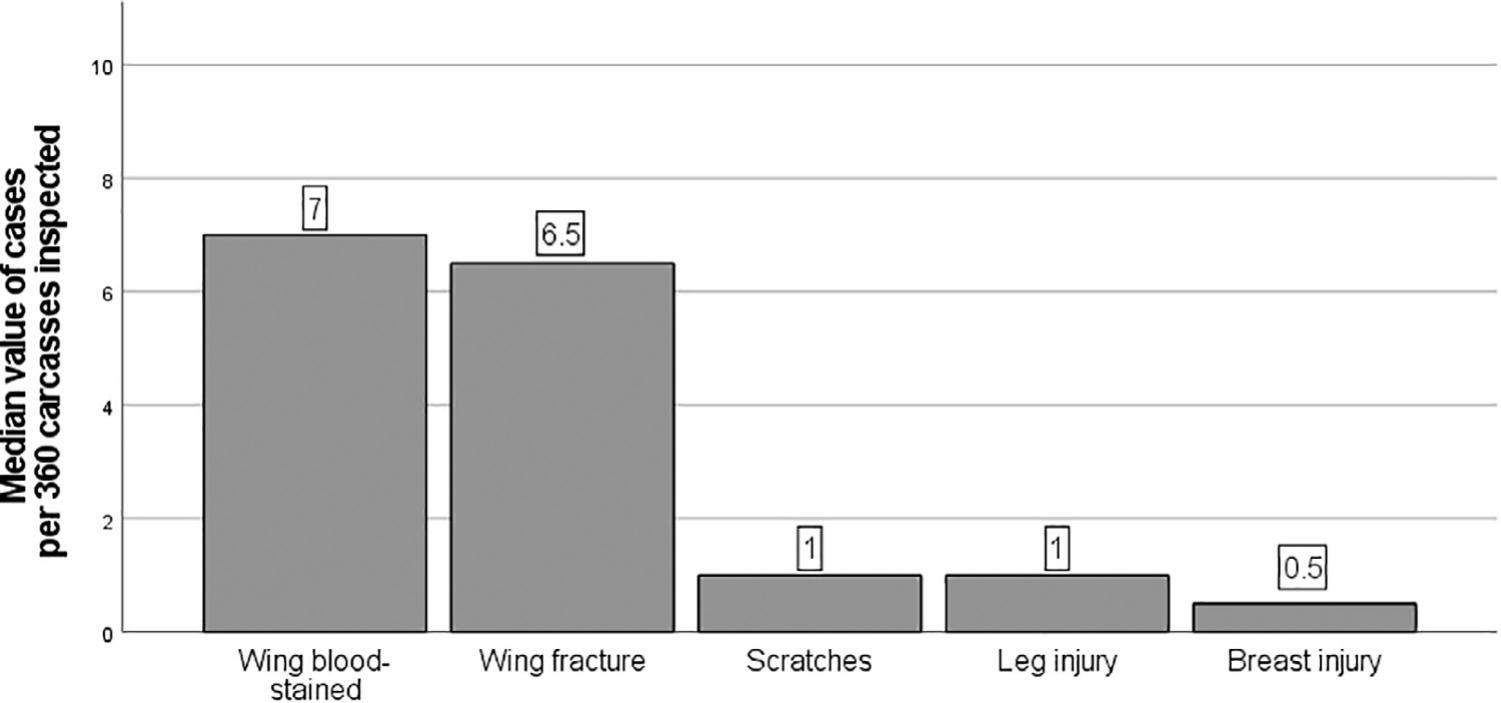
The median number of animal welfare-related findings recorded in the slaughterhouse during 3-mo period calculated based on 360 examined carcasses per flock.

Prevalence of limb fractures from the slaughterhouse were collected from 333 flocks slaughtered between 1.01.2022 and 31.03.2022. The average size of the flock (± standard deviation) was 17,060 ± 10,062 chickens. The chickens originated from 3 countries, the Netherlands, Belgium, and Germany. The top 3 most prevalent animal welfare-related findings were wings with petechiae/bruises/hematoma (wing blood-stained), wing fractures and carcass scratches/leg injury (Figure 3).

### Fracture Characterization

The results of the fracture characterization of the 143 collected samples including location of the injury are given in Table 1. Apart from samples with injuries (PRE or POST), there were 13 wings and 12 legs with no injuries (HEAL). The extended description of the samples including location (distal, midshaft, proximal), type of injury (comminuted, oblique, transverse fracture, or dislocation) and date of collection can be found in Supplementary material 1. The most frequently affected part of the wing was the elbow in both PRE and POST. In legs in POST the tibiotarsal fracture was the most prevalent pathology reported, while in PRE the most frequent observation was injury with no visible fracture.

**Table 1:** Characterization of the wings and legs with fractures included in the study.

Affected bones or joints	PRE	POST	HEAL
WINGS; affected bone/joints, *n* = 83	41	29	13
Humerus	3	2	-
Elbow	27	22	-
Ulna	5	1	-
Radius	-	1	-
Carpus/metacarpus/phalanx	-	3	-
No visible fracture but injury signs are present	6	-	-
LEGS; affected bone/joints, *n* = 60	12	36	12
Femur	-	-	-
Tibiotarsus	2	33	-
Fibula	-	-	-
Tarsometatarsus/digits	-	-	-
No visible fracture but injury signs are present	10	3	-

PRE, prekilling fractures; POST, postkilling fractures, HEAL, healthy samples (no injury).

### Interpretation of CT Scans—Level of Agreement Between Veterinarians

The level of agreement between 3 veterinarians and 1 animal welfare officer (4 raters in total) from the slaughterhouse expressed as Fleiss` kappa is given in Table 2. In all 3 rounds the same rating from the animal welfare officer was used. The values of Fleiss’ kappa in identifying the presence of fracture/dislocation (yes/no) were 0.807 and 0.827 in 1st and 2nd round, respectively. While the values of Fleiss’ kappa in determining the fracture type (PRE/POST) were 0.279 and 0.363 in 1st and 2nd round, respectively. In the 3rd round the value of Fleiss kappa was 0.663 (yes/no) in identifying the presence of the fracture/dislocation and 0.580 in determining its type (PRE/POST).

**Table 2:** The level of agreement between raters (expressed in Fleiss’ kappa) in identifying the fracture and its type.

Occurence and type of the fracture	1st round^1^ (CT scans only)	2nd round^2^ (CT scans + photographs)	3rd round^3^ (photographs only)
Occurrence of the fracture/dislocation (yes/no)*	0.807	0.827	0.663
Type of the fracture (PRE/POST)*	0.279	0.363	0.580

1 During 1st round 3 veterinarians were using CT scans only and it was confronted with animal welfare officer classification.

2 During 2nd round 3 veterinarians were using CT scans with the corresponding photographs and it was confronted with animal welfare officer classification.

3 During 3rd round 3 veterinarians were using photographs only and it was confronted with animal welfare officer classification.

⁎ For every cell with Fleiss’ kappa coefficient values the *P* value was <0.05. CT, computed tomography; PRE, prekilling fractures; POST, postkilling fractures.

### Histopathology

**Figure 4:**
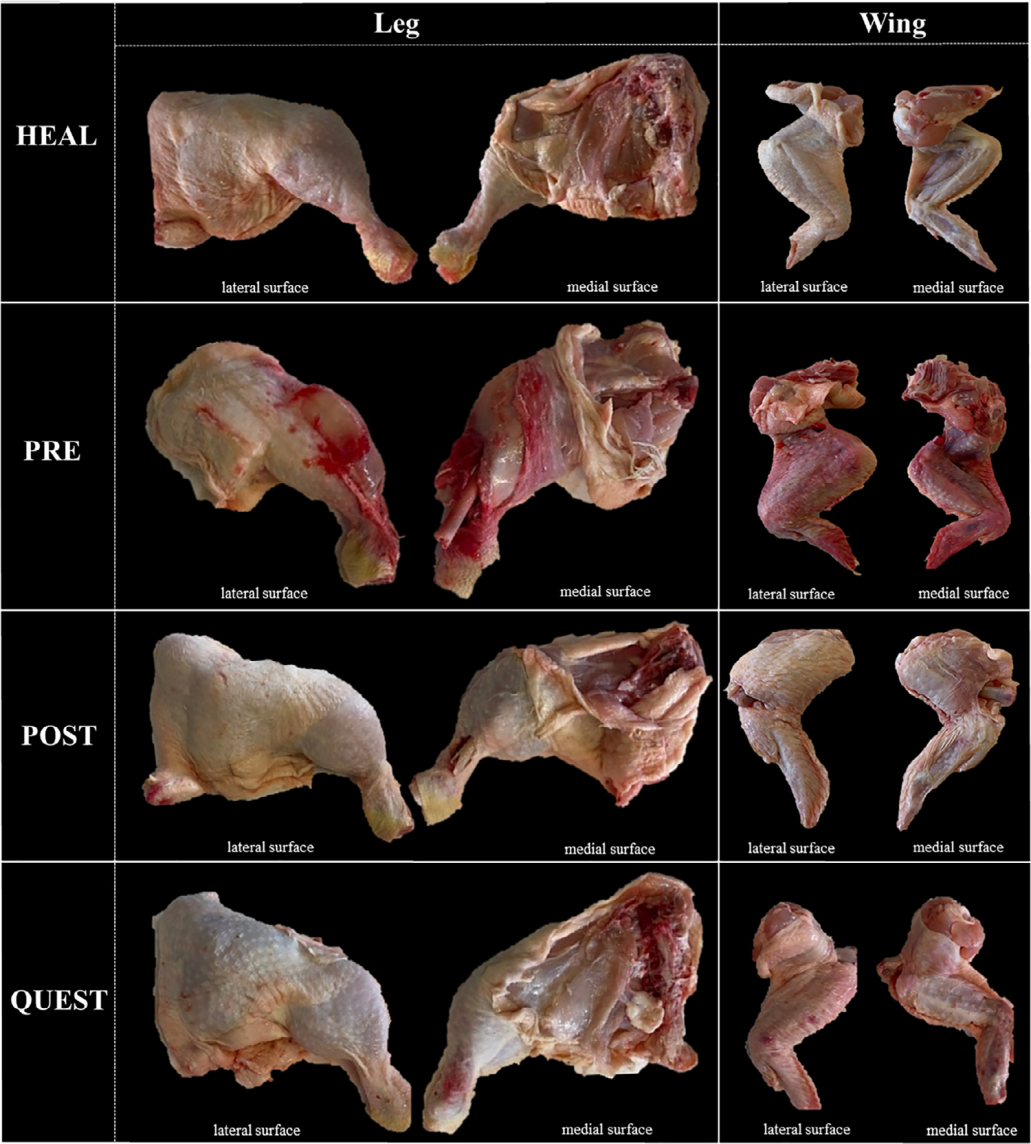
Overview of the chicken legs and wings samples included in the histopathological examination; HEAL, no injury/fracture; PRE, prekilling fracture, POST, postkilling fracture; QUEST, questionable type of sample.

Figure 4 provides an overview of the chicken wings and legs samples subjected for histopathological examination.

As expected, no pathological changes (NCh) were identified in the HEAL wings and legs. In the PRE wing and leg samples blood-staining (Bs), petechiae/bruises (PCh), non-natural bone/joint alignment (Na) and open fracture/dislocation (OFd) were observed. While in POST leg and wing samples only non-natural bone-alignment (Na) and open fracture/dislocation (OFd) were reported. Blood-staining (Bs) and petechiae/bruises (PCh) were both observed in a mild form in the QUEST category for legs and wings. The gross lesions characteristics of HEAL, PRE, POST, and QUEST are briefly summarized in Table 3.

**Table 3:** The macroscopic evaluation of the samples included in histopathological examination.

Category	Leg	Wing
HEAL	NCh	NCh
PRE	Bs, PCh, Na, OFd	Bs, PCh, Na, OFd
POST	Na, OFd	Na, OFd
QUEST	Bs*, PCh*	Bs*, PCh*

No changes (NCh); blood-stained (Bs); petechiae/bruises (PCh); non-natural bone/joint alignment (Na); open fracture/dislocation (OFd).

⁎ Lesions were reported in mild form. HEAL, healthy samples (no injury); PRE, prekilling fractures; POST, postkilling fractures; QUEST, questionable type of sample.

In the histopathological examination no pathological changes (NCh) were reported in HEAL legs and wings. In the PRE leg sample hemorrhage (H), fibrosis (F) and increased intracellular space (Is) were found, while in the PRE wing sample hemorrhage (H), necrosis (N) and granulocytic infiltration (GrI) were the main findings. In the POST leg sample fibrosis (F), necrosis (N), and mild form of granulocytic infiltration (GrI) were identified. Whereas, in the POST wing sample signs of fibrosis (F) were also found, but additionally increased intracellular space (Is) was reported. In the QUEST leg sample signs of fibrosis (F) were confirmed. Furthermore, hemorrhages (H) and granulocytic infiltration (GrI) were reported, both in a mild form. In the QUEST wing sample apart from fibrosis (F), the presence of mild form of hemorrhages (H) and mononuclear infiltration (MoI) were found.

**Figure 5:**
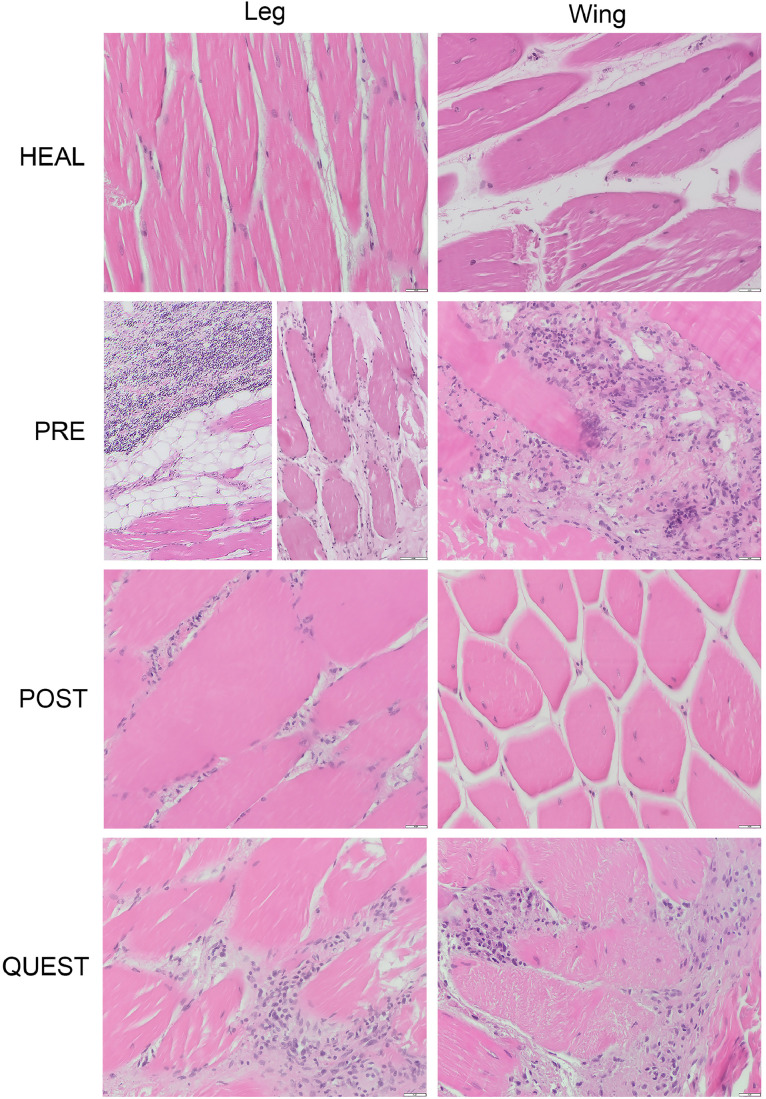
Histopathological sections of chicken legs and wings; sections stained with hematoxylin and eosin (HE), magnification 400× (PRE-leg 200×), HEAL, no injury/fracture; PRE, prekilling fracture; POST, postkilling fracture; QUEST, questionable type of injury.

The summary of histopathological findings is given in Table 4 and histopathological sections are presented in Figure 5.

**Table 4:** The summary of histopathological findings in HEAL, PRE, POST and QUEST samples.

Category	Leg	Wing
HEAL	NCh	NCh
PRE	H, F, Is	H, N, GrI
POST	F, N, GrI⁎	F, Is
QUEST	F, H⁎, GrI⁎	F, H⁎, MoI⁎

No changes (NCh); hemorrhage (H); necrosis (N); granulocytic infiltration (GrI); mononuclear infiltration (MoI); fibrosis (F), Increased Intracellular space (Is).

⁎ Lesions were reported in mild form. HEAL, healthy samples (no injury); PRE, pre-killing fractures; POST, post-killing fractures; QUEST, questionable type of sample.

## DISCUSSION

The main aim of this study was to investigate the usefulness of CT scanning in recognizing leg and wing fractures of broiler chicken and to differentiate between PRE and POST fractures. We also compared if visual inspection increases the reliability of the inspection. Based on our results the combination of CT scanning and visual inspection ensures subsequently high reliability in; 1. determining the presence of a fracture (yes/no) and 2. its type (PRE/POST). In contrast, assessment based on CT scans only results in relatively high reliability in recognizing fractures (yes/no), but lower reliability in determining its cause (PRE/POST) and assessment based on visual inspection exclusively results in relatively high reliability of determining cause of the injury (PRE/POST), but lower reliability in recognizing fractures (yes/no). Therefore, the key advantage of CT scanning (identification of bone/joints injuries) should be brought together with the merit of visual inspection (identification of gross lesions) and this combination should be further investigated and developed. It is also likely that carcass scanning and visual inspection would provide information not only limited to animal welfare, but also related to other aspects of the veterinary surveillance in the slaughterhouse, for example food safety and food quality issues.

### Analysis of Animal Welfare Inspection Reports

Limb fractures, particularly in wings, are significant animal welfare concerns in the poultry industry. These fractures cause pain and suffering (Gentle, 2011), and therefore, require close monitoring to uphold animal welfare. According to the literature, incidence of wing injuries varies between 0.0138 and 5.7% (Cockram et al., 2020; Valkova et al., 2021) and our results were in line with these values. The median incidence of wing injuries (wings blood-stained) was 7 cases/360 carcasses (≈1.94%) and 6.5 cases/360 carcasses (≈1.81%) for wing fractures. While the mean value for wing injuries was 6.69 cases/360 carcasses (≈1.86 %) and 6.33 cases/360 carcasses for wing fractures (≈1.76%). In the bar plot (Figure 3) we presented the median value instead of the mean, since the distribution of number of chickens with wing fractures was not normally distributed (*P* value <0.05). Nevertheless, if these numbers were multiplied by the number of chickens slaughtered per day (in our study the daily capacity is approx. 100,000 chickens), then it would result in considerable number of chickens (almost 2,000) with wing injuries per day. It has been established that most of the fractures occur before slaughter (PRE) (Kittelsen et al., 2015) and fractures can emerge in the rearing barn or during handling and transport. The preslaughter handling at the abattoir appears to be the critical step, in particular shackling. According to Kittelsen et al. (2015), the mean frequency of broilers with broken wings was 0.8% at the lairage and this number increases up to 2.90% after shackling just before stunning. Comparable results were presented in another study, in which the mean frequency of the broilers with broken wings was 0.99% and 1.67% at lairage and postshackling, respectively (Jong et al., 2018). Various factors can impact the number of chickens with limb fractures, such as stocking density, the method and speed of catching the birds, and the time of day during transportation (Cockram et al., 2020). Studies have shown that catching birds by their abdomen and holding them in an upright position during loading can result in fewer wing fractures compared to catching them by their legs and holding them upside down (Kittelsen et al., 2018). Similarly, a slower loading speed resulted in the lower incidence of wing injuries (Cockram et al., 2020). On the contrary, there have been studies that have shown no significant difference in the number of chickens with wing fractures when comparing manual loading and mechanical loading with chicken harvester (Mönch et al., 2020).

### Differences in Characterization of Wings and Legs Injuries

Regardless of the origin of injuries (PRE vs. POST), the severity of the cases should be also taken under consideration. Based on our analysis and experience, wing injuries are more common than leg injuries, thus we examined more wings samples compared to legs. Wings are more susceptible to injuries compared to the legs because of their relatively smaller muscle mass. The cumulative length of leg bones (drumstick and thigh) is approximately 184 mm, whereas the cumulative length of wing bones (humerus and ulna) is 152 mm. The total mass of leg muscle is around 359 g, whereas the total mass of wing muscle is 38 g (Biesiada-Drzazga et al., 2012). Therefore, it can be deduced that every 10 mm of bone is protected by approximately 20 g of muscle in legs and 2.5 g in wings. Besides, during sudden movements of the wings (i.e., flapping), the birds can injure themselves by striking other objects such as other chickens, transportation crates or parts of the slaughterhouse line during unloading, hanging or stunning. Most likely, damage to only the soft tissues is less painful for the animal, but our study found that the dominant pathology was elbow dislocation (luxation) in wings and tibiotarsus fracture in legs. We assume that these structures are the most prone to damage and/or are the most visible and thus easiest to recognize at the slaughterhouse.

### CT Scan Interpretation

Interpreting CT scan results in medical diagnostics can be challenging, as it requires broad knowledge of anatomy, tissues, and physics (Davies and Pettersson, 2002). Furthermore, it can be considered subjective, as even experienced radiologists may make different interpretations of the exact same CT scan when examined at 2 different time points (Hadied et al., 2020). However, in the current study, our aim was to only determine the presence or absence of injuries without providing a pathological description, which is typically provided by a radiologist. The level of agreement between raters (Table 2) indicates that in the 1st and 2nd round reliability in recognizing fractures was almost perfect (>0.8), while in the 3rd round it was considered substantial (0.663). Conversely, the reliability in determining the fracture type was classified as fair in the 1st and 2nd round and moderate in the 3rd round. All of calculated Fleiss` kappa values were statistically significant (*P* value <0.05) meaning that the alignment of assessors was significantly better then random assignment. In clinical sciences, where the welfare of the individual patient is of utmost importance, the minimum value of Fleiss' kappa should reach not less than 0.8 to obtain reliable diagnosis (McHugh, 2012). However, in the context of our study, which is focused on food production on an industrial scale, we believe that results indicating fair or moderate agreement can be considered satisfactory. The combination of CT scanning and visual inspection (2nd round) can be considered as a compromise to achieve relatively high reliability of assessment in both determining the presence of fracture and determining the fracture type. It is also probable that replacing human visual inspection with a computer (camera) vision system will increase consistency of the visual part of the inspection (Sandberg et al., 2023), which is planned for the further research. It is worth noting that the quality of CT scans could also be improved, which may improve the assessment of fracture type. Fleiss' kappa has also been used in studies assessing postoperative pain in dogs or assessing animal welfare in dogs (Barletta et al., 2016; Bacon et al., 2019). In poultry studies, Fleiss' kappa has been used to assess unconsciousness in broiler chickens at the slaughterhouse, with values ranging from −0.01 to 0.77 (Contreras-Jodar et al., 2022), or to assess gait in Pekin ducks as a welfare indicator, with a range of 0.47 to 0.80 (Makagon et al., 2015). Similarly, studies of the consistency of visual meat inspection in poultry have shown moderate to good agreement between inspectors (Bisaillon et al., 1988) and 77% of identical classification of the carcasses (Fries and Kobe, 1993). All the aforementioned examples highlight the issue of subjectivity in any assessment made by humans, which was also pointed out by European Food Safety Authority (**EFSA**) in the scientific opinion on inspection of poultry meat (EFSA, 2012).

### Histopathology

There are reports examining the utility of histopathology in describing injuries in animals intended for slaughter. For instance, histopathological examination has been applied to evaluate human-caused bruises in pigs at the slaughterhouse (Barington et al., 2016), lesions at the tibiotarsal region in broilers (Cavani et al., 2022) or changes after the death of cows during transportation to the slaughterhouse (Burns et al., 2014).

In our study we decided to perform histopathological examination to determine whether visual observations correlated with certain tissue reaction patterns. In the samples categorized as PRE the dominant reaction patterns were inflammatory cells infiltration, focal spots of necrosis and multifocal clusters of erythrocytes (hemorrhages). These findings indicate that blood circulation and the immune system were still functioning just after the tissue damage and therefore seem to confirm the visual PRE categorization as correct. If injury categorized as POST would be purely the result of postmortem effects of the slaughter machinery, then histologically no signs of inflammation, hemorrhage or fibrosis would be expected. Although POST samples did not show any signs of hemorrhage, as was expected, they interestingly do show fibrosis. Increase of connective tissue likely will increase rigidity of the limbs and this might predispose these limbs postmortally for slaughter procedure damage. Potentially the fibrosis could be also identified in CT images, because increase of connective tissue results in increase of radiodensity. In the current study, patterns that might indicate fibrosis (darker areas around the affected bone) were observed in CT scan of POST leg sample, but not in wing POST sample. However, to objectively compare radiodensity between samples, quantitative scale should be used (e.g., Hounsfield scale), which was beyond the scope of the current research. In QUEST samples there were some areas with mild inflammatory cell infiltration and hemorrhage, so it can be assumed that these reactions were induced in the still living animal and therefore they should be assigned to the PRE category. Ideally, CT scans could give some extra information enabling to reclassify samples from QUEST to PRE, but such features were not found in the study during interpretation.

It needs to be considered though that freezing and thawing of the samples increase the chance of tissue presenting artifacts, for example, fluid leakage, which might be confused with edema.

### Future Perspectives, Biases, and Limitations

Utilizing computer systems to automate and enhance the decision-making process for quality inspection is essential in modern food supply chains due to urgent need to optimize the supply chain, and enhance food logistics, food delivery, and food safety (Ramirez-Asis et al., 2022). Another driver for the automatization will be the growing labor shortage in the food industry including meat industry (EURES – Labor Shortages, 2022). For instance, machine learning algorithms can be used to analyze images obtained through X-ray and/or visible light imaging, thereby enabling the automatic classification of limb injuries cases by the trained model. In the literature, numerous studies have investigated the use of machine learning in interpreting CT scans for human diagnostics (Lassau et al., 2021; Vaidyanathan et al., 2022). For example, predicting the severity of COVID-19 patients based on chest CT scans is one such application. Although visible light imaging (visual inspection) is less expensive and less complex than X-ray imaging in terms of safety and implementation, it still has a significant limitation in that visual inspection cannot visualize internal structures. For example, in some cases, the limbs may appear normal from the outside despite bone fractures inside. In such cases, the use of X-ray setups such as CT scan would be advantageous over visible light imaging.

Currently, the most significant challenge is the subjectivity in distinguishing between PRE (an animal welfare issue) and POST (not an animal welfare issue) fracture types. It can be argued that even training an algorithm in the future to differentiate between PRE and POST fracture types is not free from subjectivism since humans will select the training data (scans with a given interpretation - labels). However, interpreting scans during training data selection is undoubtedly less biased than decisions made by animal welfare officers working on the production line in a hostile environment and under time pressure. Standardization of the differentiation between PRE (an animal welfare issue) and POST (not an animal welfare issue) is highly desired to establish standardized animal welfare monitoring procedures in various slaughterhouses across different countries. The count of POST can also be useful for the abattoir, as it can suggest the need for repairs and adjustments to machinery. For example, prolonged defeathering time can increase the incidence of bone fractures (Shung et al., 2022). This could motivate business operators to improve animal welfare standards (PRE) or adjust and repair the machinery (POST). Future studies should also try to determine the actual ratio of PRE and POST fractures.

The electrical stunning (electrical water-bath stunning) and controlled atmosphere stunning are 2 commonly applied stunning methods in modern poultry slaughterhouses. The slaughterhouse that provided samples for our research is using the controlled atmosphere stunning (CO_2_ stunning), which can have an impact on our reported prevalence of wing and legs fractures injuries. Therefore, it is important to determine the type of stunning method, when analyzing wing or legs injuries in chicken. For example, in another research the observed percentage of damaged wings was significantly higher (3.6 vs. 2.2%) in chickens stunned in controlled atmosphere stunning compared to electrical stunning (Riggs et al., 2023). This difference can be probably explained by excess of wings flapping leading to injuries observed in controlled atmosphere stunning (Riggs et al., 2023).

The chronology of the interpretations rounds could also potentially influence the consistency of the interpretation. In our study the order of interpretation rounds was as follows: 1st CT scans only, 2nd CT scans + photographs, 3rd photographs only. One could suggest that more logical order would be to start with CT scans only (1st round), followed by photographs only (2nd round), and then analyze CT scans + photographs in the 3rd round. The order of interpretation in our study was predetermined by the study design, because initially our aim was to interpret CT scans only, but we realized that combination of visual inspection may add extra value to the consistency (2nd round). Then we performed interpretations based on photographs only as the last round (3rd), because it is a standard method used in the slaughterhouses. Nevertheless, by ensuring long enough time interval between rounds (1- and 7-mo) we prevented the bias associated with assessors being able to memorize the samples, which could have influence their interpretations.

One limitation of our study is the moderate number of samples scanned, and that the scanning was conducted in a veterinary clinic rather than a slaughterhouse environment due to technical and financial restrictions. Another limitation is the absence of a gold-standard or reference, which usually exists in diagnostic imaging. However, there is no gold-standard available for the entire inspection process in slaughterhouses, including postmortem and animal welfare inspection. For example, in studies investigating novel methods of postmortem inspection (on-site vs. remote) or comparing inspections between official veterinarians/auxiliaries, the performance is expressed and compared in kappa coefficient (Arzoomand et al., 2019; Almqvist et al., 2023) rather than being related to a gold-standard. However, one can assume that the gold-standard in the current research would consist of thorough ante-mortem inspection (physical examination) of every bird in the lairage outside of the transporting crate to check for any signs of injuries followed by the detailed dissection of every carcass performed just after slaughter. The dissection should be preferably conducted by a veterinary pathologist rather than official veterinarian and samples for histopathology evaluation would be taken to confirm findings. However, these procedures are almost impossible to conduct in the slaughterhouse environment due to high workload, costs and high-throughput production of modern poultry slaughterhouses. Therefore, the effectiveness and quality of inspection in the current form will be always dependent on the knowledge, experience, and thoroughness of the official veterinarian or meat/animal welfare inspector.

One of the current limitations of introducing CT scan technology in the industry is the investment cost, experimental and computational time, the radiation protection and safety procedures for X-rays in an industrial environment. Therefore, currently this technology might be too complicated and expensive for many slaughterhouses in different countries. Imaging techniques using visible light (e.g., camera system) are more accessible. Nevertheless, the intensive development of CT scan technology may help to overcome some of these disadvantages in the future.

## CONCLUSIONS

The combination of CT scanning with visual inspection ensures a high reliability in both determining the presence of fracture (yes/no) and its type (PRE/POST), since it implements the key advantage of CT scanning (identification of bone/joints injuries) together with the merit of visual inspection (identification of gross lesions). In the histopathological images signs of inflammation, necrosis and hemorrhage were reported in PRE that were not found in POST. In the questionable samples (QUEST) hemorrhages were found, which indicates that these samples fit better in PRE category.

The future research will focus on CT scanning combined with the visual technique, which probably would be a visual camera due to possibility of automation. In case of questionable samples histopathological examination could be a reference method. The ultimate goal would be to design the in-line setup, which could identify limbs fracture in real time and then automatically decide to remove affected carcasses from the production line.

## Appendix Supplementary materials

Image, application 1

## Footnotes

Supplementary material associated with this article can be found in the online version at doi:10.1016/j.psj.2023.103403.
